# Presegmenter Cascaded Framework for Mammogram Mass Segmentation

**DOI:** 10.1155/2024/9422083

**Published:** 2024-08-09

**Authors:** Urvi Oza, Bakul Gohel, Pankaj Kumar, Parita Oza

**Affiliations:** ^1^ Computer Science Dhirubhai Ambani Institute of Information and Communication Technology, Gandhinagar, Gujarat, India; ^2^ Computer Science & Engineering Nirma University, Ahmedabad, Gujarat, India

## Abstract

Accurate segmentation of breast masses in mammogram images is essential for early cancer diagnosis and treatment planning. Several deep learning (DL) models have been proposed for whole mammogram segmentation and mass patch/crop segmentation. However, current DL models for breast mammogram mass segmentation face several limitations, including false positives (FPs), false negatives (FNs), and challenges with the end-to-end approach. This paper presents a novel two-stage end-to-end cascaded breast mass segmentation framework that incorporates a saliency map of potential mass regions to guide the DL models for breast mass segmentation. The first-stage segmentation model of the cascade framework is used to generate a saliency map to establish a coarse region of interest (ROI), effectively narrowing the focus to probable mass regions. The proposed presegmenter attention (PSA) blocks are introduced in the second-stage segmentation model to enable dynamic adaptation to the most informative regions within the mammogram images based on the generated saliency map. Comparative analysis of the Attention U-net model with and without the cascade framework is provided in terms of dice scores, precision, recall, FP rates (FPRs), and FN outcomes. Experimental results consistently demonstrate enhanced breast mass segmentation performance by the proposed cascade framework across all three datasets: INbreast, CSAW-S, and DMID. The cascade framework shows superior segmentation performance by improving the dice score by about 6% for the INbreast dataset, 3% for the CSAW-S dataset, and 2% for the DMID dataset. Similarly, the FN outcomes were reduced by 10% for the INbreast dataset, 19% for the CSAW-S dataset, and 4% for the DMID dataset. Moreover, the proposed cascade framework's performance is validated with varying state-of-the-art segmentation models such as DeepLabV3+ and Swin transformer U-net. The presegmenter cascade framework has the potential to improve segmentation performance and mitigate FNs when integrated with any medical image segmentation framework, irrespective of the choice of the model.

## 1. Introduction

Breast cancer remains one of the most prevalent and life-threatening diseases affecting women worldwide. Early diagnosis and treatment planning play a crucial role in reducing mortality rates. Among the multiple imaging modalities used in different stages of breast cancer treatment, mammography has been widely used to identify breast lesions, particularly masses, at an early, treatable stage [1]. However, the effectiveness of mammography hinges on the precision and reliability of mass segmentation—a critical process that identifies and isolates suspicious lesions within breast mammograms. Segmenting breast masses allows for a more precise analysis of their characteristics, such as size, shape, and density, which is critical for determining whether a mass is benign or malignant. Through the past decades, automated breast cancer computer-aided diagnostic (CAD) systems have been proposed to automate and help radiologists read and interpret mammograms for diagnosis [2, 3].

Multiple deep learning (DL) architectures have been developed to enhance the accuracy and efficiency of breast mass segmentation. Among these architectures, fully convolution network (FCN)-based models, including the U-net architecture and its variations [4–6], are commonly employed for mammogram mass segmentation tasks. The existing literature predominantly offers two kinds of segmentation models for breast mass segmentation: mass patch/crop segmentation [5, 7] and whole mammogram segmentation [8–10].

The mass patch segmentation model focuses on segmenting mass regions within localized patches of the mammogram image. It is a comparatively more straightforward task since it operates on localized areas of the breast where masses are suspected or detected. While effective in segmenting breast mass regions with a deficient number of missed mass pixels outcomes, the mass patch segmentation models are not end-to-end models and often require additional detection networks to detect the presence of breast masses from mammograms to extract the region of interests (ROIs) for patch segmentation model training/testing [7, 11]. Thus, mass patch segmentation models are typically used as a component of an integrated framework where masses are localized using detection models and later segmented using patch segmentation models [12–15]. Integrated frameworks for breast cancer diagnosis have been proposed by combining different components/tasks, such as mass detection, segmentation, and classification, into a single unified framework [14–16]. These frameworks include sequential processing, where images pass through a series of models one after another to simultaneously perform both breast mass detection and segmentation in a single pass. However, handling false positives (FPs) and false negatives (FNs) remains a significant challenge with such integrated models, as additional processing is required before providing them to segmentation models. Here, FP refers to cases where normal breast tissues are mistakenly segmented as breast masses, and FN refers to cases where actual breast masses are missed or not segmented by the segmentation models. The patch segmentation models tend to predict FP results when tested as an end-to-end model on randomly extracted mammogram patches from all over the breast regions rather than just breast mass detected regions by the detection models. The whole mammogram segmentation models aim to segment the breast mass from the entire image. However, as they process mammograms as the whole image as a single entity without focusing on specific ROIs where masses are more likely to occur, it can cause the model to overlook subtle mass regions, leading to FN results.

Cascade frameworks, also known as cascaded models, are a series of machine learning models or stages designed to make sequential decisions about the input data. Like integrated frameworks, cascade models consist of multiple stages, each designed to perform a specific subtask in the overall medical imaging process. However, integrated models have interdependent tasks within the same architecture, while cascaded models operate with a sequential dependency between stages. These cascaded frameworks are increasingly used in various medical imaging applications [17–19]. Cascaded frameworks have been proposed on mammogram images for preprocessing and mass detection in the initial stage, followed by segmentation in the subsequent stage [20, 21]. Considering the limitations of existing whole mammogram and mass patch segmentation models, we propose a novel two-stage cascaded framework integrating both of these models for breast mass segmentation. Our proposed cascaded framework uses a modified patch segmentation model in the first stage and whole mammogram segmentation as the second-stage segmentation model. This innovative approach allows us to combine the strengths of both patch-based and whole mammogram segmentation models while mitigating their respective weaknesses. As the patch segmentation models tend to have less FN and more FP results, we used their output to generate a saliency map for potential mass regions to guide segmentation in the next stage. Whole mammogram segmentation models in the second stage, guided by the output saliency map generated in the previous stage, would further perform segmentation with refined output and fewer FN and FP results. Generally, in cascaded frameworks, the input image is processed sequentially through stages, where the output of one stage serves as input to the next stage, and so on. However, in order to better guide segmentation in the second stage, we propose passing the output of the first stage, that is, the saliency map, to the proposed presegmenter attention (PSA) blocks rather than directly providing them to the second-stage segmentation model. Ultimately, we propose a cascade framework with the goal of developing a more accurate and reliable segmentation framework for breast mass diagnosis, thereby improving patient outcomes and advancing the field of medical imaging.

We propose a cascade framework for whole mammogram mass segmentation tasks, where sequential models are used with the aim of optimizing precise segmentation. Our objective is to progressively refine segmentation results, from identifying probable mass regions to leading to better mass segmentation. The hierarchical nature of the proposed cascade framework for mass segmentation enables error correction in segmentation and the identification of subtle masses that may be missed in single-pass segmentation methods. We incorporated the proposed cascaded framework with the Attention U-net segmentation model [22]. Moreover, we have also extended our research by including the results of the proposed cascaded framework with recent segmentation architectures such as DeepLabV3+ [23] and Swin transformer models [24]. These models were selected based on their popularity and effectiveness in medical image segmentation tasks. Here, Attention U-net represents models that incorporate attention mechanisms into traditional architectures like U-net to improve the model's focus on informative image regions. DeepLabv3+ represents a class of convolutional neural network (Convnet)-based segmentation models that leverage atrous convolution and spatial pyramid pooling to capture multiscale contextual information effectively. In contrast, the Swin transformer represents the emerging trend of transformer-based architectures in image segmentation, which utilize self-attention mechanisms to capture long-range dependencies and global context. We also provide insights into how our proposed framework contributes to accurately identifying and segmenting benign and malignant breast masses.

The main contributions of this paper are as follows: (1) a two-stage cascade framework for breast mass segmentation. The first stage model, called “presegmenter,” segments potential mass regions from entire mammograms to establish a coarse ROI and narrow the focus to relevant areas, laying the foundation for more accurate segmentation in the second stage. We emphasize patch-based segmentation rather than whole image segmentation as a presegmenter model. (2) Introducing the PSA block to guide attention to potential mass regions in the second-stage segmentation model. Based on the output saliency map of the presegmenter model, the PSA block dynamically adapts to the most informative regions within mammogram images. Moreover, it can be seamlessly integrated with any advanced encoder–decoder architecture for the segmentation model, providing flexibility and adaptability to different research and clinical settings. (3) Presenting a comprehensive evaluation of the proposed cascade framework on various segmentation models to highlight the efficacy and generalizability of the proposed framework across different datasets and clinical scenarios.

## 2. Related Work

The appearance of breast masses can vary significantly in size, shape, and texture, making their segmentation more challenging than the relatively uniform objects typically found in natural images. In the context of breast mass segmentation, several DL models and methods have been proposed, including U-net [6, 11], FCNs [17], conditional random field (CRF) models [25], and Attention U-net [9, 26], among others. Researchers have employed various methods to train models for segmentation on mass patches extracted from breast mammograms. These include obtaining mass-centered patches by extracting a rectangle bounding box around manually annotated mass centers. Various techniques have been explored to determine the optimal cropping approach, such as tight ROIs focusing only on the lesion within the bounding box [5], ROIs with padding relative to the mass area (height and width increased by a defined percentage) [25, 27, 28], or fixed windows around the lesion [7, 11]. However, one limitation shared by these methods is that extracting mass patches is typically a manual process, ensuring the presence of a mass at the center of the input for segmentation models. In pursuit of fully automated CAD systems, Dhungel, Carneiro, and Bradley proposed a model where masses were detected, segmented, and later classified using an integrated DL framework [16, 29]. Similarly, an automatic DL model based on You Only Look Once (YOLO) was employed for mass detection, providing mass patch patches for segmentation by models like the full-resolution convolutional network (FrCN) [15] and connected U-nets [14]. In these integrated frameworks, the input for mass segmentation depends on the detection model's output. It is worth noting that models trained on manually extracted ROIs tend to outperform those relying on bounding boxes detected by detection models, highlighting the significance of patch extraction as a critical step for patch-based mass segmentation. The output of detection models may have mistakenly detected the normal tissue as mass (FP), which requires further preprocessing of detected ROIs before feeding them into the segmentation model. Currently, most frameworks require manual exclusion of detected FP ROIs before the segmentation stage, which may not be considered a fully automated end-to-end approach [14, 15, 29]. Thus, rather than the detection and segmentation of mass patches, some segmentation models have been trained on whole mammogram images to predict the binary segmentation mask of a given mammogram. However, mammogram images' inherent complexity and variability make segmenting masses from entire mammograms a formidable task. The authors have noted the difficulty in distinguishing tumors from nontumor regions due to the high similarity in intensity value distributions [30]. Khamparia et al. [31] proposed a hybrid transfer learning model as an effective tool to reduce such errors, especially FNs/missed tumors, in the clinical setting. Integrating attention mechanisms within encoder–decoder architectures has also been widely adopted to encourage models to focus on informative areas, such as masses. Sun et al. incorporated attention gates (AGs) and dense connections into the traditional U-net to enhance segmentation accuracy [9]. Abdelhafiz et al. introduced RU-net by stacking residual attention modules onto the basic U-Net architecture [32]. Another approach, the attention-guided dense upsampling network (AU-net) with an effective attention-guided dense upsampling block (AU block) and asymmetric encoder–decoder structure, was designed for accurate mass segmentation [9]. Researchers have utilized visual saliency maps to guide attention in ultrasound image features for segmentation, making identifying regions more likely to attract radiologists' visual attention [33]. Ning et al. also used saliency maps incorporating low-level and high-level image structures to guide lesion segmentation in breast ultrasound images [34]. Attention modules empower segmentation models to focus on local details within and around breast masses, improving boundary delineation and reducing segmentation errors.

Cascade models have been introduced to tackle various segmentation challenges in medical imaging data. Cascade frameworks designed for both detection/localization and segmentation typically consist of multiple stages or models that operate sequentially. For example, Luo et al. proposed a two-stage cascaded deep neural network for adrenal gland segmentation in an end-to-end fashion, where the first-stage localization network was used to determine the target organ and the second-stage segmentation network was used to refine the boundary [17]. Similarly, cascaded frameworks have been proposed for the detection and segmentation of pulmonary nodules [35], the detection of the whole heart, and accurate segmentation of the heart substructures [36]. In contrast, certain cascade frameworks are tailored exclusively for segmentation tasks. For example, Zhao et al. proposed a two-stage cascade segmentation strategy for tooth segmentation scenarios on low-contrast dental panoramic X-ray images [19]. This approach employed global and local attention modules to roughly localize dental regions in the first stage and an FCN to precisely identify dental regions in the second stage. Jiang et al. also proposed a two-stage cascaded U-Net to segment the substructures of brain tumors from coarse to fine [18]. Different cascade stages were tailored to address specific challenges related to the imaging subject. Cascade segmentation models generally perform ROI segmentation in the first stage, and precise boundary segmentation is performed on the segmented ROI in the second stage model [37–39]. The first-stage ROI segmentation results or presegmenter results can guide the segmentation process for better performance. Such segmentation-cascaded frameworks have been proposed to guide the segmentation of tumors in ultrasound images by utilizing saliency maps generated from first-stage models [33, 34]. In the proposed work, we follow a similar approach and propose the two-stage cascaded segmentation framework for mammogram mass segmentation. Rather than detection and segmentation cascade models, we propose segmentation-focused cascade models that directly tackle the segmentation task based on the presegmenter output.

## 3. Methodology

Our proposed framework is built upon the U-net architecture [40], a well-established encoder–decoder framework commonly employed for segmentation tasks. The U-net comprises two main components: the encoder and the decoder. In the encoding phase, the spatial dimensions are successively reduced by a factor of two at each scale, facilitating the extraction of abstract image features. In contrast, the decoding phase leverages the encoder's features to predict a segmentation mask, eventually restoring it to the original image size. However, this reduction in spatial dimension during encoding can result in losing vital spatial context. Thus, extracted features from the encoder levels are passed as skip connections and concatenated to the respective decoder-level features to help retrieve better localization information. These skip connections help learn spatial information while restoring the features needed to predict the segmentation mask in the decoder path. This traditional U-net model architecture has been modified by integrating AGs instead of skip connections to get more effective segmentation results [22]. The Attention U-net model was proposed to learn to focus on target structures of varying shapes and sizes for medical image segmentation tasks. AGs filter the features propagated through the skip connections. Feature selectivity in AGs is achieved using contextual information (gating) extracted on coarser scales. These features are then upsampled in the decoding part of the network to predict the binary segmentation mask as an output.

While both AGs and skip connections contribute to better feature selectivity in the decoder, they do not leverage additional information about potential mass regions within the mammogram. The proposed cascade framework leverages prior knowledge obtained from the first-stage segmentation model, called the “presegmenter model,” to guide the second-stage mammogram segmentation. We hypothesized that this integration enhances the second-stage model's ability to differentiate between mass and nonmass regions, reducing both FNs and FPs. Current whole mammogram segmentation models, including U-net variants, typically lack this level of contextual guidance. We propose a “presegmenter attention” (PSA) block to guide encoder feature extraction and propagation via skip connections. We have implemented the proposed cascade framework with Attention U-net as a segmentation model.

### 3.1. Presegmenter Model

**Figure 1:**
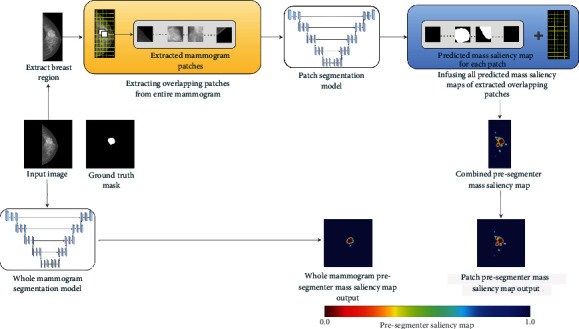
Generating presegmenter saliency map (visualized as color heat map) from (i) patch segmentation model: the U-net model trained on mass patches is used to segment overlapping patches from an entire mammogram; predicted mass saliency maps are aggregated to generate a final saliency map and (ii) whole mammogram segmentation models: the U-net model trained on whole mammogram images is used to predict mass the saliency map of a given mammogram.

The first stage of the cascade framework utilizes a presegmenter model to compute a saliency map indicating potential mass areas within the input mammogram. This saliency map is a crucial auxiliary input for the subsequent encoder stages in the second-stage segmentation model. To facilitate this process, we introduce the PSA block, which harnesses the presegmenter saliency map to apply spatial attention at each encoder level of the second-stage segmentation model. This attentive mechanism employs the presegmenter saliency map, which is scaled down across all encoder layers, focusing attention on regions of the mammogram with high-intensity values in the saliency map. Once the mammogram image is passed through the preprocessing pipeline, we will calculate the presegmenter saliency map by passing it to the presegmenter model. We considered two configurations for the presegmenter model: the patch segmentation model and the whole mammogram segmentation model.  Figure 1 illustrates a pipeline of the proposed cascade framework's first-stage segmentation model/presegmenter model. The figure shows how patch segmentation and whole mammogram segmentation models can generate presegmenter saliency maps.

#### 3.1.1. Patch Presegmenter Model

For the patch presegmenter model, we trained a U-net–based model specifically tailored for the segmentation of mammogram mass patches in an end-to-end framework. We provided manually extracted mass patches to train the segmentation model to generate its respective segmentation masks. Considering the size of breast masses present in the dataset and exploiting the maximum possible resolution, we extracted a patch of size 1024 × 1024 centered on the breast mass. This ensures capturing the entire mass of variable sizes in the center of the patch to train the patch segmentation model. In case the breast mass is located at the corner or at the edge of the image, we employed padding to extend the mammogram and mask patch to a size of 1024 × 1024 to ensure comprehensive coverage. The number of mass patches extracted from each mammogram was contingent on the number of masses present within the ground truth segmentation mask. These extracted patches and their segmentation masks were resized to 256 × 256 before serving as input to train the patch segmentation model. Notably, as part of an end-to-end framework, we did not possess prior knowledge of the mass locations required for patch extraction during the testing phase. Therefore, we extracted overlapping patches spanning the entire test mammogram image to accommodate this challenge. To avoid missing any mass (if localized near the edge or corner of the patches) while extracting patches, we decided to extract overlapping patches with 50% overlap from the test image. So, given a mammogram of size *N* × *N*, we extracted patches of size 1024 × 1024 with a stride of 512 between patches from the cropped breast region. Let us denote *P* as the set of patches extracted from the mammogram. These overlapping patches were then passed through the trained patch segmentation model. For each patch from set *P*, denoted as *P*_*i*_, the patch segmentation model will predict the mask, denoted as *M*_*i*_, where each pixel indicates the probability value (ranges between zero and one) of it belonging to breast mass. We may apply thresholding to these predicted probability values to generate a binary segmentation mask. However, we are using these masks with probability values between zero and one as saliency maps to indicate pixels with probable mass regions. Thus, after passing all patches through the segmentation model, we obtain a set of predicted mass saliency maps, {*M*_1, *M*_2, ⋯, *M*_*k*}, where *k* is the total number of extracted patches. We combined these patch saliency maps to construct the final mass saliency map for the entire mammogram image and employed the max operation on overlapping pixel regions. Similarly, a logical OR operation was used to generate a combined binary segmentation mask of a given mammogram by combining the binary segmentation masks of patches. We opted for this approach based on its ability to capture potential mass regions identified by the patch segmentation model from any subsequent overlapping patches. Even if mass pixels are detected in only one patch, they will be included in the final combined saliency map or binary segmentation mask, thereby reducing the chance of FNs. The final patch presegmenter saliency map denoted as *M*_final_ for the entire mammogram would be of size *N* × *N* after padding it to the original input image size, as shown in Figure 1. According to prior studies, the patch segmentation models demonstrate robustness in correctly identifying and accurately segmenting patches containing genuine masses. Thus, the culmination of the mass saliency map *M*_final_ effectively identifies potential mass regions within the mammogram image.

#### 3.1.2. Whole Mammogram Presegmenter Model

Alternatively, we employed a U-net model trained on the whole mammogram instead of a mass patch for mammogram mass segmentation as the presegmenter model. The whole mammogram segmentation model will predict the mask for the entire mammogram, where each pixel indicates the probability value (ranging from zero to one) of it belonging to breast mass. As can be seen from Figure 1, this predicted output mass saliency map for the given mammogram can be directly utilized as the presegmenter output.

### 3.2. Segmentation Model

After passing through the presegmenter model, the mammogram image is passed to the second-stage segmentation model of the proposed cascaded framework along with the generated presegmenter saliency map. We considered the commonly used encoder–decoder architecture for the second-stage segmentation model, such as Attention U-net [22]. The Attention U-net architecture has demonstrated state-of-the-art performance in various medical image segmentation tasks, including breast mass segmentation. Its architecture is known for its ability to focus on relevant regions of an image, which aligns with the goal of our cascaded framework to highlight potential mass regions identified by the presegmenter model. By integrating the presegmenter output as spatial attention, the Attention U-net can dynamically adapt its segmentation process to focus on these highlighted regions, thereby improving overall segmentation performance.

Both mammogram images and their respective saliency maps of size *N* × *N* will be resized to 512 × 512 before feeding into the Attention U-net model. The mammogram image *X* ∈ *R*^3×512×512^ initially undergoes encoder levels, consisting of convolution and max pool layers, to extract informative features that later pass to subsequent encoder levels. Our proposed method integrates the extracted features from the previous level into the proposed PSA block, where guided features from the PSA block output are transmitted to the next encoder level.

**Figure 2:**
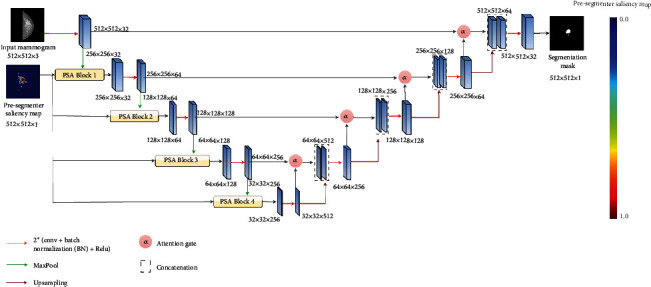
Architecture of the proposed cascade framework with the PSA block–guided Attention U-net model as a second-stage segmentation model for mammogram mass segmentation.

Figure 2 shows the architecture of the Attention U-net (second-stage segmentation model of the proposed cascade framework) with proposed PSA blocks integrated at each encoder level. As shown in Figure 2, in addition to these PSA blocks, an AG is applied at each decoding stage of Attention U-net to combine features from the encoder with those from the decoder. Encoder and decoder features serve as inputs to the AG, passing through convolution layers before undergoing element-wise summation. This process assigns higher weights to aligned features and lower weights to misaligned ones. The resulting vector undergoes ReLU activation and convolution layers, culminating in attention coefficients computed via sigmoid activation. These coefficients signify the relative importance of each spatial location in the encoder feature map with respect to the corresponding location in the decoder feature map. The computed attention coefficients are multiplied element-wise with the encoder feature maps, emphasizing regions deemed important by the attention mechanism. We trained the Attention U-net model with the Adam optimizer and dice loss as a loss function (Equation (1)).

(1) $$\begin{array}{c} \text{Dice loss }\left(p,g\right)=1-\frac{2\sum_{i}^{n}p_{i}g_{i}}{\sum_{i}^{n}{p_{i}}^{2}+\sum_{i}^{n}{g_{i}}^{2}+\epsilon \;} \end{array}$$

Here, *p*_*i*_ and *g*_*i*_ represent pairs of corresponding prediction and ground truth pixel values, respectively, and *ε* is a smoothing factor added to avoid dividing by zero error. We trained the model with a learning rate of 10^−4^ and a batch size of two. All these DL models are trained using TensorFlow and Keras libraries on the NVIDIA GeForce RTX 3060 GPU (12 GB RAM).

#### 3.2.1. PSA Block

The motivation behind introducing the PSA block is to guide feature extraction and strengthen the significance of features extracted from potential mass areas at each encoder level. Our PSA block is influenced by spatial attention mechanisms introduced in the convolutional block attention module (CBAM) [41] and the AGs designed to guide the model's focus toward critical regions [22]. Spatial attention mechanisms adaptively recalibrate feature maps, while AGs selectively gate information flow in U-net–like architectures. In the context of CBAM, a spatial attention module was proposed to emphasize informative portions of an image based on interspatial relationships among image features. This was achieved by applying max pool and average pool operations on image features, followed by concatenation and convolution, yielding a 2D spatial attention map [41]. In the proposed PSA block, inspired by the spatial attention mechanism, a presegmenter saliency map was used to focus on relevant regions of interest within the mammogram images, effectively guiding the segmentation process to areas with potential masses highlighted by the presegmenter model. Inspired by the AG mechanism that facilitates the selective integration of features, we utilize the presegmenter saliency map as a gating signal to guide extracted encoder features from the previous stage to the next encoder stage based on the derived spatial attention coefficients.

**Figure 3:**
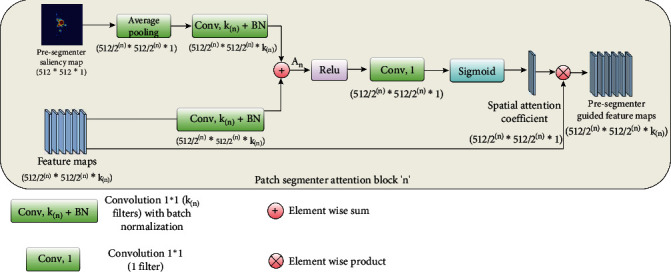
Architecture of the PSA block: Input to the block is a presegmenter saliency map of size 512 × 512 × 1 and encoder feature maps of size 512/2^*n*^ × 512/2^*n*^ × *k*_*n*_ for the PSA block at layer level *n*, with *k*_*n*_ number of channels.

Figure 3 illustrates a diagram of the PSA block. It comprises two inputs: one being the presegmenter saliency map *P*_*n*_ and the second being the encoder feature maps *F*_*n*_ at a particular encoder stage *n*. We considered the segmentation model with five levels/stages. The convolution filters per level are 32, 64, 128, 256, and 512, respectively. The input feature maps to the PSA block are denoted as *F*_*n*_′ = {*F*_1_, *F*_2_, ⋯, *F*_*kn*_}, where each feature map *F*_*n*_ has spatial dimensions of 512/2^*n*^512/2^*n*^ × *k*_*n*_ for the block at layer level *n*, where *n* ∈ {1, 2, 3, 4, 5}. Here, *k*_*n*_ represents the channel dimensions of encoder feature maps in block *n*, denoted as *k*_*n*_ = {32,64,128,256,512}. For example, the PSA block at Level 1 would entail encoder feature maps with dimensions of 256 × 256 × 32 and a presegmenter saliency map measuring 512 × 512 × 1.

For the PSA block at encoder level *n*, the presegmenter saliency map from the previous stage undergoes downsampling through an average pool layer to align with the input spatial dimensions of the encoder feature maps. So, the presegmenter saliency map *P*_*n*_ at level *n* would be

 $$\begin{array}{c} P_{n}=\text{AvgPool }\left(P_{n-1}\right) \end{array}$$

where the output saliency map from the presegmenter model can be represented as *P*_0_ ∈ *R*^1×512×512^. Once both inputs *P*_*n*_ and *F*_*n*_ share comparable spatial dimensions, they are linearly mapped through a combination of a convolution layer with a channel size of *k*_*n*_ and a batch normalization layer. These features from both inputs are then fused via element-wise addition, represented as *A*_*n*_.

 $$\begin{array}{c} {\text{A}}_{\text{n}}=\left({\text{f}}_{\text{kn}}^{1\times 1}\left({\text{P}}_{\text{n}}\right)+{\text{f}}_{\text{kn}}^{1\times 1}\left({\text{F}}_{\text{n}}\right)\right) \end{array}$$

Here, *f*_*kn*_^1×1^(.) denotes a convolution operation with a *k*_*n*_ number of filters and 1 × 1 kernel size, followed by batch normalization.

After applying the ReLU activation function, the fused feature descriptor *A*_*n*_ is reduced to a single channel using a 1 × 1 convolution, generating a spatial attention mask. The sigmoid activation function is subsequently applied to normalize all values from zero to one, representing the normalized spatial attention coefficients. The final output of the PSA block materializes as an element-wise multiplication of the obtained spatial attention coefficients with each channel of encoder feature maps *F*_*n*_. So, the presegmenter-guided feature map (*O_n_*) is

 $$\begin{array}{c} O_{n}=\sigma \left(f^{1\times 1}\left(\text{RELU}\left(A_{n}\right)\right)\right)⊗F_{n} \end{array}$$

where *σ* denotes the sigmoid function, ⊗ denotes elementwise multiplication, and *f*^1×1^ represents a convolution operation with the Filter Size 1 and kernel size 1 × 1. These presegmenter-guided feature maps *O*_*n*_ are then provided as a gating signal to the corresponding decoder block, effectively highlighting crucial mass regions within the image. Moreover, these guided features extend their influence to the next encoder layer, further reinforcing the model's attention on pertinent areas.

## 4. Experiment

### 4.1. Dataset

Our study leveraged three distinct digital mammography datasets: INbreast [42], CSAW-S [43], and DMID [44]. We employed high-resolution mammograms encompassing both the mammography views: mediolateral oblique (MLO) and craniocaudal (CC). Below, we provide a comprehensive overview of each dataset:

• INbreast. This widely recognized public dataset is a cornerstone in breast mass segmentation research. We employed all mammograms presenting mass abnormalities in both mammogram views, totaling 107 images. The dataset provided binary ground-truth mass segmentation maps for each mammogram. Due to its limited image count, we employed a five-fold cross-validation strategy to train and assess the proposed model's performance.
• CSAW-S. It is a subset of the larger CSAW dataset, the most extensive public mammographic dataset available. While the original CSAW dataset contains mammogram images without segmentation masks, the CSAW-S dataset, a fully annotated subset of CSAW, provides a sufficiently diverse and representative sample of mammogram images along with segmentation masks. CSAW-S offers 312 training and 26 test mammograms, each accompanied by semantic segmentation annotations. Each patient's mammogram is enriched with segmentation masks comprising 12 distinct pixel-level label annotations, meticulously annotated by an expert radiologist. These 12 labels encompass tumor, calcification, thick nerves, nipple, skin, pectoral muscle, nonmammary tissue, mammary glands, foreign objects, auxiliary lymph nodes, text, and unclassified objects. As our focus is on mass segmentation, we exclusively considered pixel labels pertaining to tumors, designating all other labels as background or nonmass. The evaluation of our trained models was conducted using the test data provided by the dataset.
• DMID. We incorporated a recently published dataset named DMID for our experiments. For every abnormal image in the collection, corresponding binary masks are provided. The dataset boasts over 500 images showcasing various pathologies, including benign, malignant, microcalcification, asymmetry, and distortion. We selected 267 mammograms featuring benign and malignant mass abnormalities for the study. To ensure robustness, we employed a five-fold cross-validation approach to train and test the segmentation models on this dataset.

### 4.2. Data Preprocessing

We passed the mammogram dataset through the following preprocessing pipeline to remove artifacts, normalize, and resize the images before providing them as input to the model.

• Flipping. Given that the dataset contains left and right breast mammograms with varying orientations, we applied a flipping operation to align all mammograms in the same orientation uniformly.
• Background artifact removal. The largest contour in the mammogram (i.e., breast region) was detected and cropped as the rectangle box to remove extra background artifacts and pixels.
• Padding. After removing the background artifacts, we pad the mammograms with background pixels. Most Convnets expect square images with equal width and height as input. Thus, we applied padding to ensure the mammogram attained the requisite dimensions. Our earlier experiment results show that resizing input images to the DL model's input layer dimensions will not change the aspect ratio if we have square images. Additionally, padding protects against potential cropping during augmentation techniques such as rotation or translation, which might inadvertently affect the breast area.
• Resizing. All the preprocessed images and masks were resized to 512 × 512 dimensions to train the model.
• Normalization. We employed a truncation normalization and enhancement procedure, a well-established practice in DL-based mammogram studies [45–48]. Within the breast region, we identified the minimum intensity value at the fifth percentile and the maximum intensity value at the 99th percentile from the sorted pixel intensity distribution. These identified values were used to truncate pixel intensities within the breast region, effectively mitigating noise and abnormal values. Subsequently, the truncated pixel values within the breast region were normalized using the min–max normalization technique. To enhance the image, we applied the Contrast Limited Adaptive Histogram Equalization (CLAHE) [49] method, further improving the contrast of breast masses. For enhanced feature extraction by the model, we synthesized a three-channel image comprising the truncated and normalized image alongside a contrast-enhanced image, with clip limits set at 0.01 and 0.02, as recommended by Cao et al. [45]. Additionally, breast segmentation masks underwent scaling to map background pixel values to zero and mass pixel values to one.
• Augmentation. To augment the dataset, we applied various operations, including rotation (30°), translation (−0.2 to +0.2), and flipping. These augmentations were crucial for enhancing the model's robustness and ability to generalize to different orientations and translations of mammograms.

### 4.3. Evaluation

We used the following evaluation metrics to evaluate the performance of the proposed models.

• Dice similarity coefficient (DSC). It measures the overlap between predicted and ground truth mass pixels.

 $$\begin{array}{c} \text{DSC}=\frac{2*\left|\text{GT}\cap \text{PR}\right|}{\left|\text{GT}\right|+\left|\text{PR}\right|} \end{array}$$

Here, |*GT*| and |*PR*| are the total number of pixels in the ground truth and predicted segmentation masks, respectively, and |*GT*∩*PR*| is the number of positive pixels/mass pixels predicted correctly.

• Recall/true positive rate (TPR). It is the ratio of the sum of correctly identified mass pixels and actual mass pixels. It represents the proportion of mass pixels in the ground truth that were correctly segmented by the model.

 $$\begin{array}{c} \text{TPR}=\frac{\text{TP}}{\text{TP}+\text{FN}} \end{array}$$

• Precision. It is the ratio of the sum of correctly predicted mass pixels to the total mass pixels. It measures the quality of the predictions/segmentation by the model.

 $$\begin{array}{c} \text{Precision}=\frac{\text{TP}}{\text{TP}+\text{FP}} \end{array}$$

• FP rate (FPR). It is the ratio of the number of FP (breast pixels or background pixels predicted as mass) pixels and actual nonmass/breast pixels.

 $$\begin{array}{c} \text{FPR}=\frac{\text{FP}}{\text{FP}+\text{TN}} \end{array}$$

• FN outcomes. We calculate the FN outcomes of the model by calculating a number of mammograms for which the entire mass is missed, and the model cannot predict a single pixel from the entire breast mass. For such cases, DSC would be considered zero, as no mass is predicted. The number of FN cases from a total number of mammograms (in terms of percentage) is provided while evaluating performance.

## 5. Results

**Figure 4:**
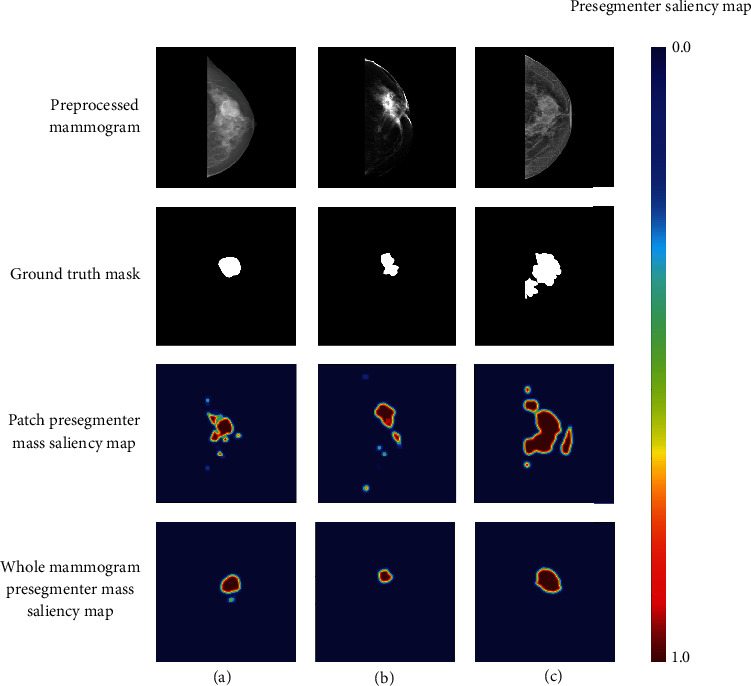
Presegmenter saliency map generated for mammograms from different datasets: (a) INbreast dataset (b) CSAW-S, (c) DMID.

**Figure 5:**
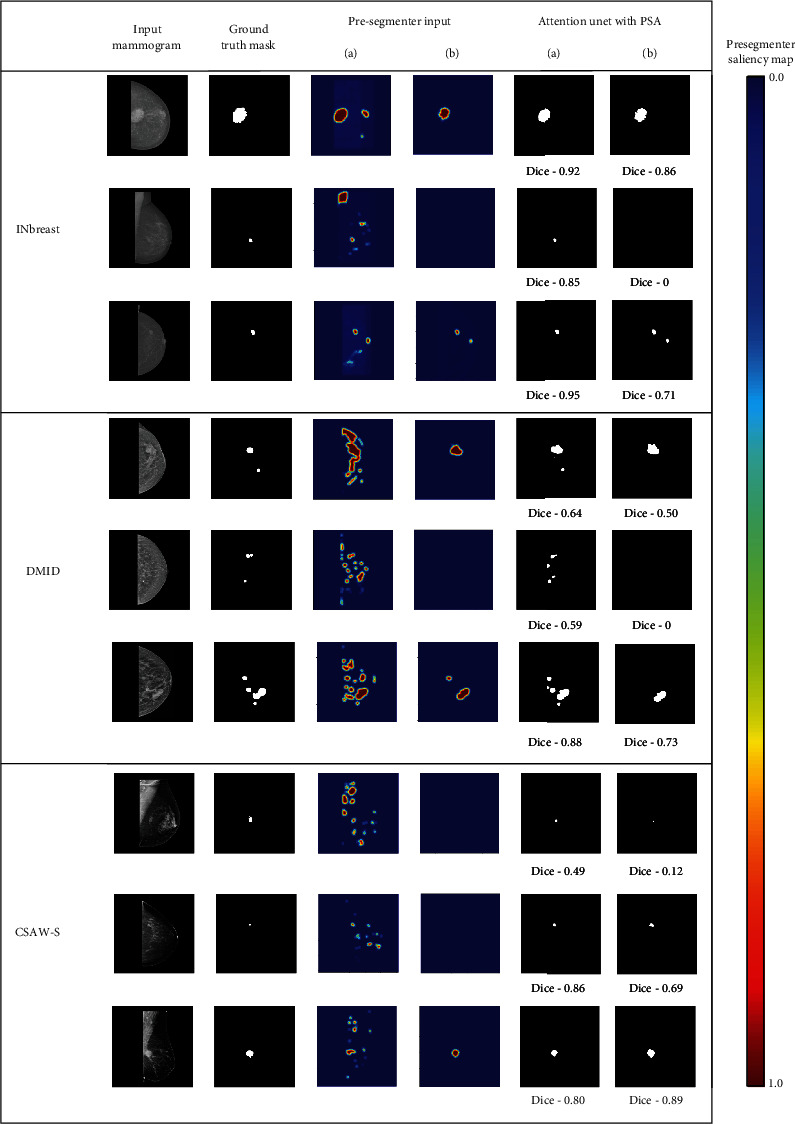
Segmentation results by Attention U-net with PSA block for two kinds of PSA inputs: (a) patch presegmenter saliency map and (b) whole mammogram presegmenter saliency map.

In this section, we begin by presenting the results obtained from commonly used mammogram segmentation models available in the literature, including both whole mammogram segmentation and mass patch segmentation, which were evaluated as part of an end-to-end framework. Subsequently, we proceed to showcase and validate the effectiveness of our proposed cascade framework with Attention U-net models.  Table 1 displays the results achieved by the trained U-net models for both whole mammogram and mass patch segmentation. Evaluation metrics were applied at both image and patch levels for the patch-based U-net segmentation model. At the patch level, we calculated evaluation metrics exclusively based on the ground truth mass segmentation mask and the predicted binary mass segmentation mask. Conversely, the evaluation metrics were computed using the ground truth and the combined segmentation outputs (binary masks) of overlapping mammogram patches for the image-level analysis. As illustrated in Table 1, the patch-based segmentation model attained the highest dice scores when evaluated on manually extracted mass patches. However, it is noteworthy that its dice score decreased and the FPR increased when tested on overlapping patches from the entire image (image-level). In contrast, the whole mammogram segmentation models demonstrated lower FPR values but recorded a significantly higher number of FN.  Figure 4 illustrates examples of presegmenter saliency maps generated by both of these models for mammograms from different datasets. As can be seen from Table 1 and Figure 4, the patch segmentation model performs exceptionally well on mass tumor patches without any FN cases. Thus, when the saliency map generated by the patch segmentation model is used as input for the PSA block, it effectively enhances the performance of Attention U-net models, as demonstrated in Table 2. Attention U-net models experienced significant improvements in recall, dice scores, and reduction in FN outcomes when coupled with the proposed cascade framework. To determine whether the whole mammogram segmentation or patch segmentation model is to be used as a presegmenter model, we conducted an experiment by employing the saliency map output from both these models as input to the PSA block in the second-stage segmentation model (Attention U-net).  Table 3 compares the results achieved using both types of PSA inputs, revealing that the PSA block performed notably better with the patch segmentation model output than with the whole mammogram segmentation model. For a comprehensive visual representation of the outcomes produced by both of these models, please refer to Figure 5.

Breast masses can exhibit significant variations in their characteristics, including shape, size, and density. Some masses may be well-defined and spherical, while others might be irregular or elongated. These differences in mass characteristics can influence segmentation models. Therefore, to ensure the clinical relevance of the proposed framework across diverse patient populations and clinical scenarios, we present the evaluation scores for benign/noncancerous and malignant/cancerous masses in the INbreast and DMID datasets (Table 4). As all mammograms in the CSAW-S dataset fall under the category of malignant masses, they are not included in this table.

**Figure 6:**
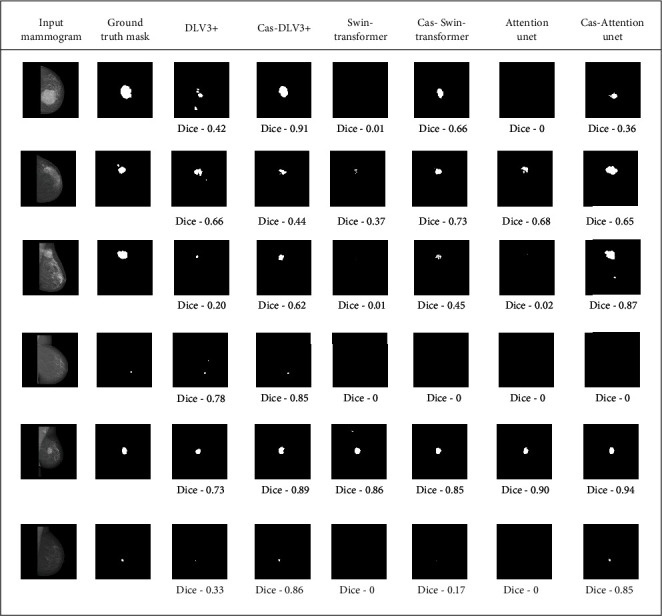
Segmentation results comparison of DeepLabV3+ (DLV3+), Swin transformer, and Attention U-net without and with proposed cascade framework with patch presegmenter saliency map on INbreast mammogram images.

In addition to evaluating the proposed cascaded framework with the Attention U-net architecture, we conducted experiments with other state-of-the-art segmentation models, including DeepLabV3+ and Swin transformer U-net. These models represent varying approaches to segmentation commonly used in medical imaging tasks. For the purpose of meaningful comparisons with existing results in the literature, we chose to limit this comparative analysis to the INbreast dataset. The INbreast dataset is a well-established public dataset widely used for breast mass segmentation studies, ensuring consistency and allowing for reliable benchmarking.  Table 5 shows the results of DeepLabV3+ (with and without pretrained ResNet50 backbone) and Swin transformer U-net with and without the proposed cascade framework, along with the Attention U-net model. As can be seen from Table 5, the performance of all these models has improved by incorporating the proposed cascade framework with a patch presegmenter saliency map. The visual results of these models with and without cascade framework are presented in Figure 6.  Table 6 compares the proposed framework results with other methods/models available in the literature on the INbreast, CSAW-S, and DMID datasets. We compared our results with the five-fold cross-validation scores achieved by other models on the INbreast and DMID datasets. For the CSAW-S dataset, scores on the provided test dataset were compared with those of other models available in the literature. As provided in Table 6, the proposed framework has comparable results with other models in terms of dice scores when tested on the same grounds.

## 6. Discussion

Segmentation is a critical step in various medical imaging applications. Cascade frameworks with presegmenter models have significantly enhanced segmentation accuracy by sequentially refining the segmentation output [38, 56, 57]. Studies have consistently shown that cascade frameworks produce more accurate segmentation results than single-stage models [58]. In this work, we present a novel cascade framework for breast mammogram mass segmentation. The presegmenter model of the proposed cascade framework detects potential mass regions at a localized level to reduce the risk of missing tumors. At the same time, the subsequent second-stage segmentation model provides a broader, global context of the entire mammogram, and helps refine the segmentation output. As a result, the proposed framework improves the segmentation performance of Attention U-net models, as seen in Table 2. Similar to the performance improvement achieved by cascade frameworks on other tasks [17, 19, 33], the proposed framework improved the breast mammogram mass segmentation results in terms of dice score and recall and reduced the number of FN cases for other segmentation models, too. The results of varying segmentation models with our proposed cascade framework (Table 5) suggest that it can be integrated with any segmentation framework, irrespective of the choice of model. Moreover, as demonstrated by Table 4, the cascade framework improves segmentation performance for both benign and malignant masses, effectively reducing FN cases for both mass classes. However, reducing FN malignant masses has more severe clinical consequences as they could delay cancer diagnosis.

**Figure 7:**
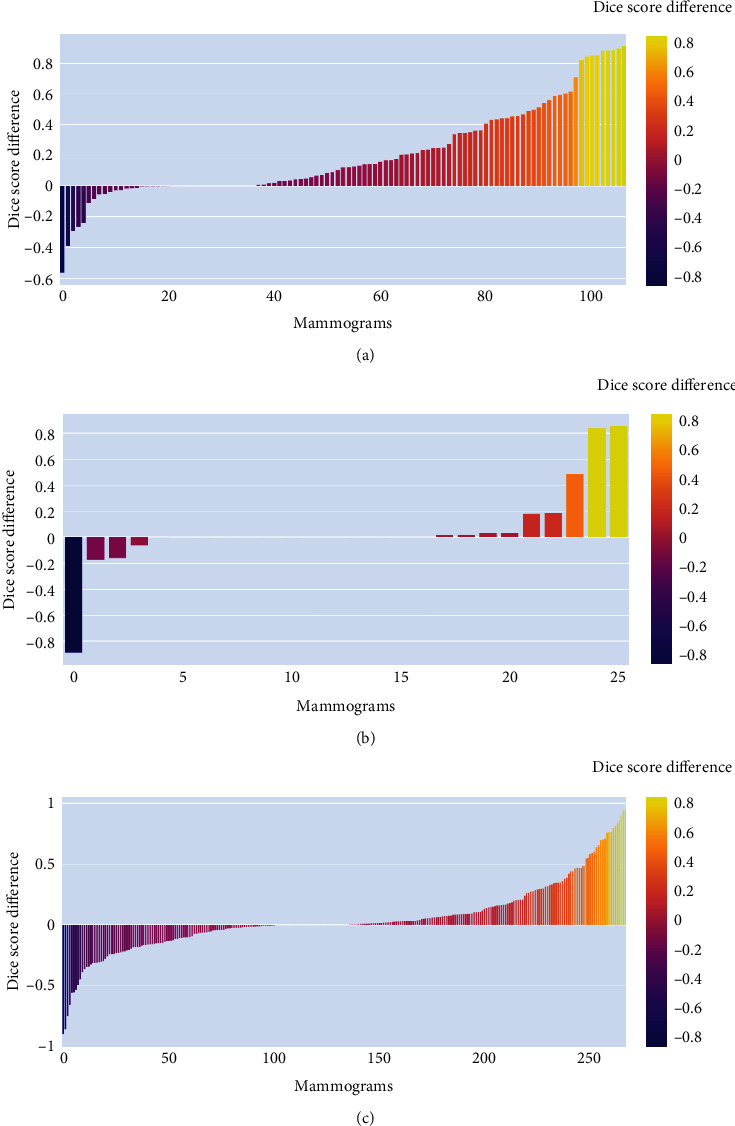
Bar graph of dice score improvement achieved by proposed cascaded framework with Attention U-net model on individual mammograms: (a) mammograms of INbreast dataset, (b) mammograms of CSAW-S test dataset, and (c) mammograms of DMID dataset.

The cascade frameworks used by prior studies utilize the output of the first-stage segmentation model as input to the second-stage segmentation model, gradually refining the segmentation results [18, 19, 57]. Many studies have used similar DL model architectures for multiple stages of cascade frameworks [59, 60]. In the proposed framework, the first stage segmentation model plays a crucial role in detecting probable mass regions to provide input to subsequent stage models. Thus, in order to choose which model to use for the presegmenter, we experimented with two approaches—the whole mammogram segmentation model and the patch segmentation model. Mass patch segmentation models are tailored to focus on smaller regions or patches within mammograms that are more likely to contain breast masses. Consequently, when assessed on manually extracted mass patches, these models achieved higher dice scores and, importantly, zero FN cases across all three datasets, as shown in Table 1. However, when these models were subjected to overlapping patches extracted from entire mammograms, the dice score decreased significantly, and FPR surged. It is evident that while these patch segmentation models excel when mass regions are present in the test input patch, they tend to predict FP for other patches, which, when aggregated, leads to lower dice scores. In contrast, whole mammogram segmentation models displayed better dice scores and lower FPR but grappled with many FN cases (Table 1). If there is an FN result from the presegmenter model, the saliency map will not be able to guide the attention of the second-stage segmentation model. Thus, the choice between employing the patch segmentation model or the whole mammogram segmentation model as a presegmenter model significantly impacted breast mass segmentation performance for the proposed framework. As shown in Table 3, the saliency map generated by the patch segmentation model used as a PSA input demonstrated better dice scores for both the INbreast and CSAW-S datasets. For the DMID dataset, the patch presegmenter saliency map as a PSA input exhibited no improvement in dice score, but it has substantially reduced the FN cases. The reduced number of FN cases by the proposed cascade framework for all three datasets suggests that the localized focus of the patch model complemented the segmentation model by aiding the identification of subtle mass regions that could be overlooked during the processing of the entire mammogram. In Figure 5, we showcase the Attention U-net model segmentation results for both types of presegmenter saliency maps as PSA inputs. It becomes apparent that while the whole mammogram presegmenter saliency map as a PSA input has less FP pixels compared to the patch presegmenter saliency map, it did not enhance but degrade segmentation performance due to increased FN cases and imprecise mass boundary delineation. Further, to substantiate the performance improvements facilitated by the proposed framework, we plotted the differences between dice scores for individual mammogram outputs generated by the proposed Attention U-net cascade framework and Attention U-net models for all three datasets (refer to Figure 7). These comparisons revealed a positive difference for a majority of mammograms across all three datasets, with a notable 67.29% of mammograms from the INbreast dataset showing improved dice scores by the proposed framework. For the CSAW-S and DMID datasets, 46.15% and 54% of mammograms, respectively, exhibited improved dice scores.

The comparative analysis with segmentation models revealed that the proposed cascade framework consistently outperformed standalone models in terms of segmentation performance. As shown in Table 5, for all considered segmentation models, the cascade framework with patch presegmenter saliency map demonstrated superior performance. Recent studies on breast cancer segmentation models show that DeepLabV3+ had comparable performance with Attention U-net models [61–63]. As DeepLabV3+ uses pretrained ResNet50 for feature extraction, the effect of transfer learning improves the segmentation results compared to Attention U-net models (as shown in Table 5). The results of DeepLabV3+ with and without pretrained ResNet50 [64] weights when integrated into the proposed cascade framework are presented in Table 5. The DeepLabV3+ without a pretrained backbone shows significant improvement in dice score, similar to the Attention U-net model when integrated with the cascade framework. Similarly, we also observed improvements in segmentation performance for DeepLabV3+ with a pretrained ResNet50 backbone when used with the cascade framework. This finding underscores the efficacy of our cascade framework in enhancing the segmentation capabilities of the model, regardless of whether pretrained weights were utilized. While pretrained models are commonly employed to leverage transfer learning and enhance performance [9], the obtained results demonstrate that even without pretraining, DeepLabV3+ benefits from the guidance provided by the proposed cascade framework. These findings contribute to the growing body of evidence supporting the effectiveness of cascade frameworks in improving segmentation outcomes across diverse model architectures. As shown in Figure 6, the proposed cascaded framework can help segmentation models detect and segment breast masses that might have been missed or not precisely segmented by the standalone segmentation models (Rows 1, 3, and 6). Results in Row 4 illustrate examples where both the traditional segmentation model and our proposed cascade framework failed to segment breast mass. These instances highlight the inherent challenges associated with breast mass segmentation, particularly when dealing with very small-sized masses.

DL models in medical image analysis, including breast mass segmentation models, were primarily focused on improving overall accuracy and performance using metrics like dice score, precision, and recall [9, 26, 30]. Nevertheless, the critical need to mitigate FN cases remains paramount, as these cases have profound implications for early and accurate breast cancer diagnosis. By minimizing FNs, our framework aims to increase sensitivity, thereby ensuring that breast cancer is detected at an early and treatable stage.

Given the widespread use of DL models in medical imaging tasks, the proposed cascade framework with a presegmenter model holds significant potential for applications beyond breast mass segmentation. Its adaptability and effectiveness in addressing segmentation challenges can be extended to other medical imaging domains, contributing to advancements in segmentation performance.

## 7. Conclusion

This paper proposes a novel cascaded breast mass segmentation framework leveraging a saliency map of the potential mass regions for the challenging task of breast mass segmentation. The saliency map generated by the patch presegmenter model is used to guide the PSA blocks to provide attention during the second stage of the segmentation model. The proposed cascade framework allows the DL model to narrow the focus to relevant areas, laying the foundation for more accurate segmentation with a reduced number of missed masses in the second stage. The proposed framework is validated with the popular segmentation models, and the results establish improved performance. Our extensive experimental evaluations across multiple datasets, including the INbreast, CSAW-S, and DMID datasets, demonstrate the superior performance of the proposed cascade approach compared to standalone segmentation models. The significance of this study lies in the presegmenter model's mechanism and the cascade framework's capability to capture intricate details of breast mass and boundary delineation, offering contextual guidance for enhanced segmentation performance, a feature often absent in many standalone segmentation models.

## Figures and Tables

**Table 1:** Results of whole mammogram segmentation model (W) and patch segmentation models (P) for all three datasets.

**Dataset**	**Model**	**Input**	**Dice**	**FPR**	**FN (%)**
INbreast	W	Whole mammogram	0.57	0.0016	22%
	P	Mass patches	0.88	0.0057	0
		Overlapping mammogram patches	0.47	0.0403	0
CSAW-S	W	Whole mammogram	0.59	0.0003	61%
	P	Mass patches	0.73	0.0309	0
		Overlapping mammogram patches	0.20	0.1270	0
DMID	W	Whole mammogram	0.65	0.0042	9%
	P	Mass patches	0.80	0.0950	0
		Overlapping mammogram patches	0.21	0.3948	0

**Table 2:** Comparison of results of Attention U-net model with and without proposed PSA block (with patch presegmenter saliency map).

**Dataset**	**Method**	**Attention U-net**				
		**Dice**	**Precision**	**Recall**	**FN (%)**	**FPR**
INbreast	Without PSA	0.65	0.86	0.65	28%	0.0006
	With PSA	0.71	0.77	0.71	18%	0.0020
CSAW-S	Without PSA	0.61	0.71	0.54	50%	0.0006
	With PSA	0.64	0.71	0.59	31%	0.0008
DMID	Without PSA	0.64	0.78	0.55	11%	0.0031
	With PSA	0.66	0.77	0.65	7%	0.0062

**Table 3:** Comparison of the proposed cascade framework with Attention U-net model for different PSA input—saliency maps generated from patch segmentation model and whole mammogram segmentation model.

**Dataset**	**PSA input**	**Attention U-net**				
		**Dice**	**Precision**	**Recall**	**FN (%)**	**FPR**
INbreast	Patch presegmenter saliency map	0.71	0.77	0.71	18%	0.0020
	Whole mammogram presegmenter saliency map	0.62	0.81	0.64	31%	0.0010
CSAW-S	Patch presegmenter saliency map	0.64	0.71	0.59	31%	0.0008
	Whole mammogram presegmenter saliency map	0.53	0.53	0.56	31%	0.0012
DMID	Patch presegmenter saliency map	0.66	0.77	0.65	7%	0.0062
	Whole mammogram presegmenter saliency map	0.66	0.64	0.76	17%	0.0045

**Table 4:** Comparison of segmentation performance of Attention U-net (AU) and proposed cascade framework with Attention U-net (Cas-AU) on benign and malignant mass classes for INbreast and DMID datasets.

**Dataset**	**Method**	**Benign mass**				**Malignant mass**			
		**Dice**	**Precision**	**Recall**	**FN (%)**	**Dice**	**Precision**	**Recall**	**F** **(%)**
INbreast	AU	0.57	0.76	0.50	32%	0.74	0.85	0.77	25%
	Cas-AU	0.62	0.63	0.64	23%	0.75	0.86	0.77	15%
DMID	AU	0.52	0.65	0.33	15%	0.69	0.94	0.60	8%
	Cas-AU	0.61	0.62	0.45	9%	0.71	0.95	0.63	8%

**Table 5:** Comparison of segmentation performance of proposed cascade framework on varying segmentation frameworks including results from both whole mammogram and patch presegmenter saliency map as PSA input trained on INbreast dataset.

**Model**	**Method**	**Dice**	**Precision**	**Recall**	**FN (%)**	**FPR**
DeepLabV3+	Without cascaded framework (without pretrained ResNet50)	0.69	0.72	0.70	11%	0.0019
	Without cascaded framework (with pretrained ResNet50)	0.77	0.81	0.78	10%	0.0014
	With patch segmentation cascaded framework (without pretrained ResNet50)	0.74	0.77	0.77	8%	0.0016
	With patch segmentation cascaded framework (with pretrained ResNet50)	0.80	0.84	0.78	8%	0.0012
	With whole mammogram segmentation cascaded framework (with pretrained ResNet50)	0.73	0.83	0.68	15%	0.0009
Swin transformer	Without cascaded framework	0.63	0.72	0.60	10%	0.0020
	With whole mammogram segmentation cascaded framework	0.60	0.69	0.59	10%	0.0019
	With patch segmentation cascaded framework	0.68	0.70	0.71	8%	0.0032
Attention U-net	Without cascaded framework	0.65	0.86	0.65	28%	0.0006
	With whole mammogram segmentation cascaded framework	0.62	0.81	0.64	31%	0.0010
	With patch segmentation cascaded framework	0.71	0.77	0.71	18%	0.0020

**Table 6:** Comparing the proposed cascade framework with Attention U-net (Cas-AU), Swin Transformer (Cas-Swin Transformer), and DeepLabV3+ (Cas-DeepLabV3+) with other methods/models available in the literature on INbreast, CSAW-S and DMID datasets.

**Dataset**	**Method**	**Metric (dice)**
INbreast	Fusion Net [50]	0.62
	FCDenseNet103 [51]	0.43
	AU-net [9]	0.64
	YOLO-LOGO [52]	0.69
	Mammo-SAM [53]	0.75
	Cas-AU	0.71
	Cas-Swin transformer	0.68
	Cas-DeepLabV3+	0.80

CSAW-S	U-net+GAN augmentation [54]	0.40
	DEEPLAB3-RESNET50 [55]	0.48
	DEEPLAB3-DEIT-S [55]	0.49
	Cas-AU	0.64

DMID	U-net [44]	0.60
	YOLOv8-based detection and segmentation [44]	0.61
	Cas-AU	0.66

## Data Availability

The datasets used in our study—INbreast and DMID—are publicly available. The INbreast dataset can be found at https://academictorrents.com/details/ce1ecade37814701ac95193a910a3c6917ea43b3. The CSAW-S dataset can be requested to access by filling out the form available at https://zenodo.org/records/4030660#.X2HD15MzZhE. The DMID dataset can be found at https://figshare.com/articles/dataset/_b_Digital_mammography_Dataset_for_Breast_Cancer_Diagnosis_Research_DMID_b_DMID_rar/24522883.
